# Supplementary material for: Live-seq enables temporal transcriptomic recording of single cells
Source: Nature. 2022 Aug 17;608(7924):733–40. doi: 10.1038/s41586-022-05046-9 (PMC9402441; doi:10.1038/s41586-022-05046-9)
Supplement: Supplementary file 1 — Supplementary Figs. 1–8, Note and Refs. [file 41586_2022_5046_MOESM1_ESM.pdf]

nature portfolio

https://doi.org/10.1038/s41586-022-05046-9

Supplementary information

Live-seq enables temporal transcriptomic recording of single cells

In the format provided by the authors and unedited

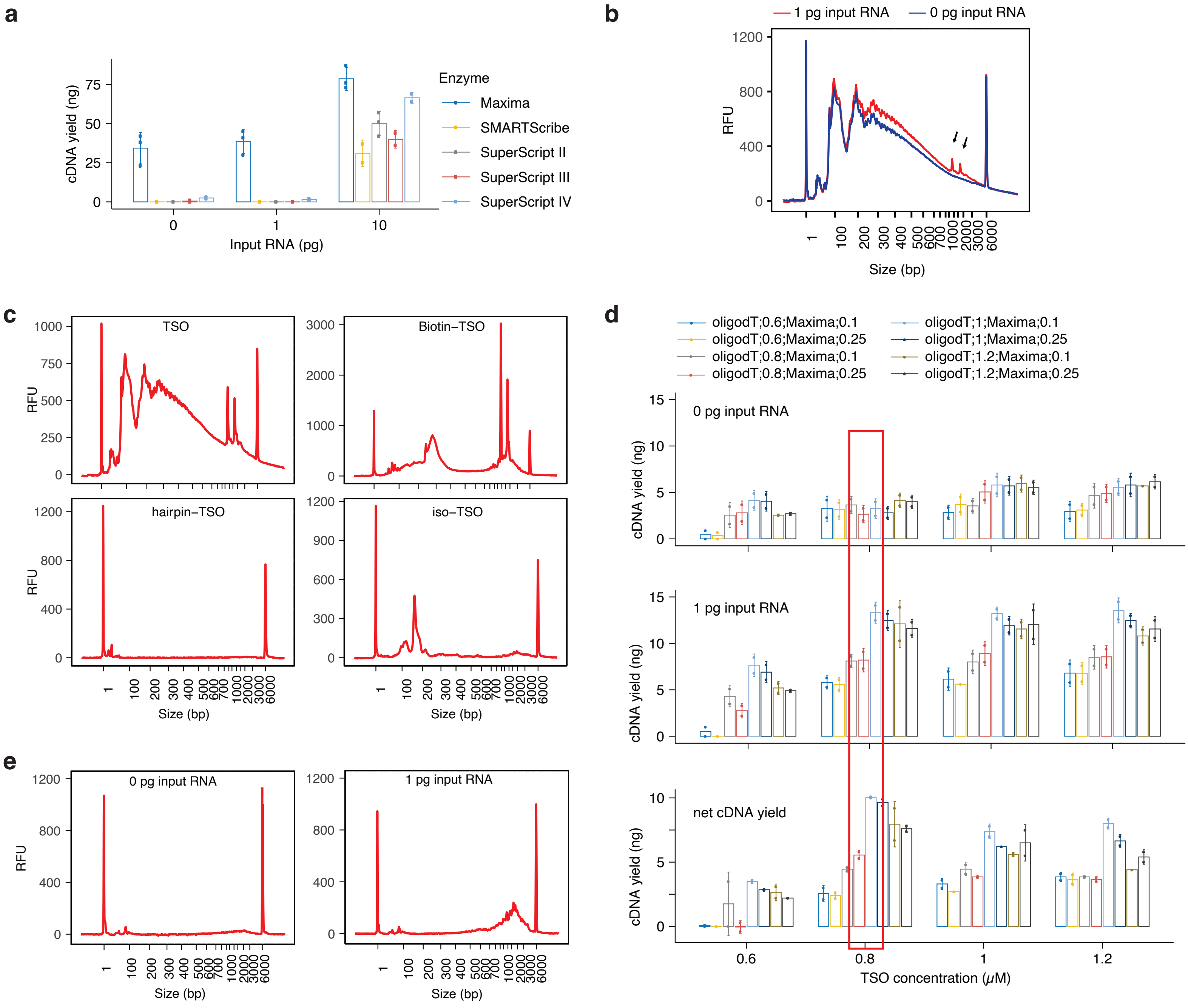

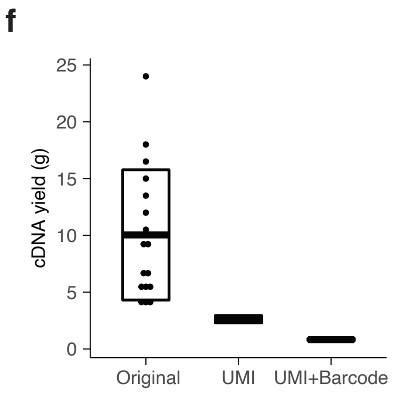

Supplementary Fig. 1. Enhanced-Smart-seq2 method development, relative to Fig. 1. (**a**) Screening of distinct reverse transcriptases, as listed. 0 (negative control), 1, and 10 pg of total RNA was used as input. N = 3 replicates. (**b**) The profile of cDNA using Maxima H- reverse transcriptase. The hedgehog pattern from 100 – 3000 bp (with peaks from 100 to 500 bp) reflects the presence of oligo concatemers. The arrows show two unique peaks in cDNA from 1 pg RNA compared to the negative control (0 pg input RNA), suggesting that bone fide signal is masked by concatemers. (**c**) Profiles of cDNAs that were generated using different 5' modified TSO primers. Maxima H- reverse transcriptase was used in all conditions. The condition using Biotin-TSO shows a high cDNA yield between 500 to 3000 bp with a relatively low amount of oligo dimers or concatemers (the hedge-hog-like pattern with peaks from 100 to 500 bp). (**d**) cDNA yields of distinct experimental set-ups involving different combinations of Biotin-TSO, oligo-dT, and reverse transcriptase from 0 pg input RNA (upper panel) and 1 pg input RNA (middle panel). The net cDNA yield was calculated as the yield from 1 pg of input RNA minus signal stemming from 0 pg of input RNA (lower panel). The workflow with the highest net yield is highlighted using a red rectangle and forthwith termed "enhanced Smart-seq2". N = 3 replicates. (**e**) Profiles of cDNA derived from the enhanced Smart-seq2 protocol. (**f**) The cDNA yield derived from 5 pg input RNA using original, UMI-bearing and (UMI + Barcode)-containing oligo-dTs.

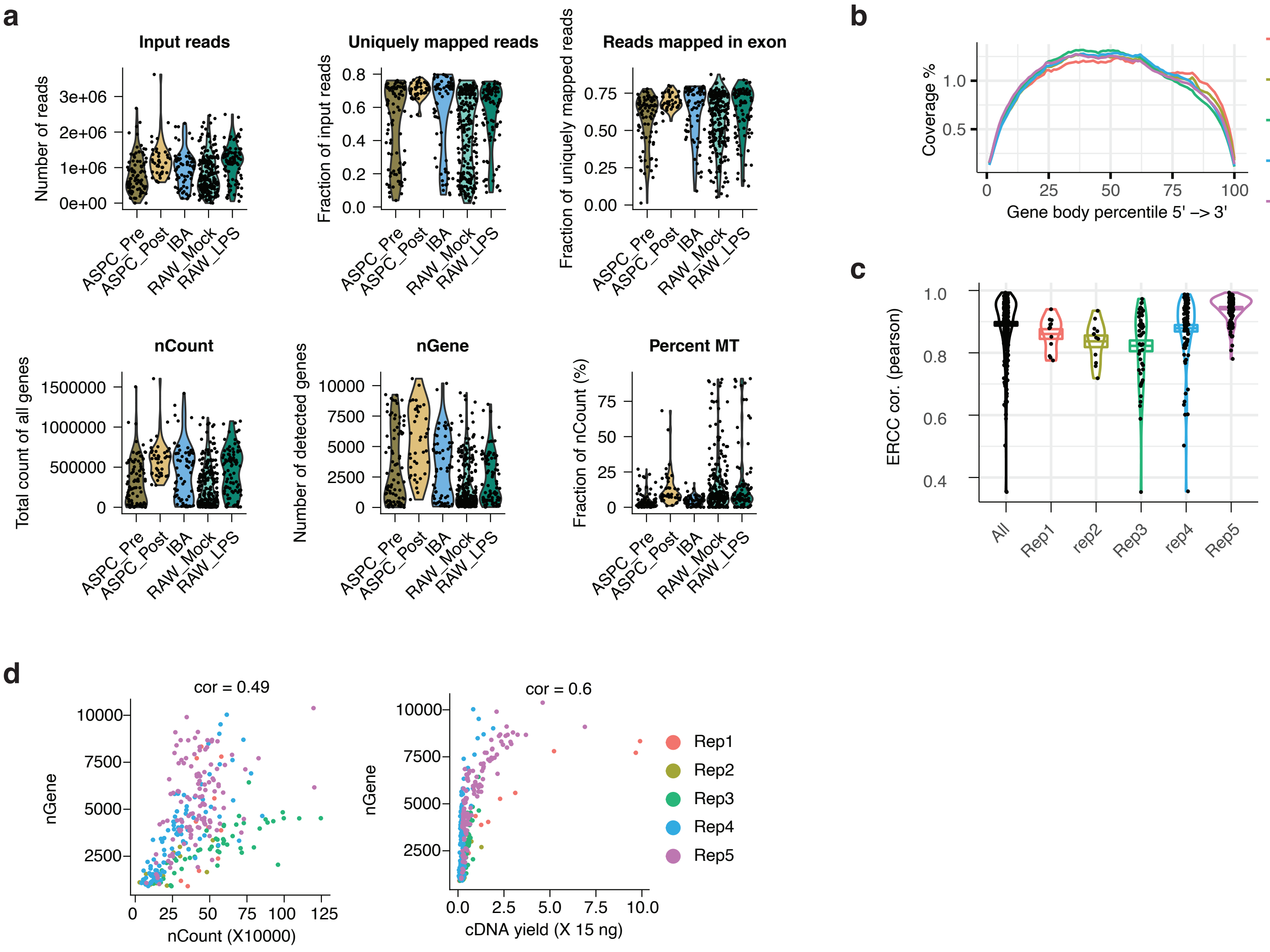

Supplementary Fig. 2. Additional quality controls of Live-seq data, relative to Fig. 2. (**a**) Number of input reads (input reads), the rate of reads uniquely mapped to the genome (uniquely mapped reads), the fraction of reads mapped to exons (Reads mapped in exon), total counts of all genes (nCount), number of detected genes (nGene) and the percentage of counts from mitochondrial genes (percent MT), for all 588 Live-seq samples/libraries are shown per cell type/state. N = 5 replicates, a total of 588 cells. (**b**) The read distribution along the body of housekeeping genes for the samples passing our quality control across different sample replicates (Rep1, Rep2, Rep3, Rep4, Rep5). (**c**) The correlation between the detected and expected amount of ERCC in each sample that passed the quality controls. The result is shown with all samples (All) or grouped by each replicate. (**d**) Plotted correlations between the number of detected genes on the one hand and respectively the total count of all genes (nCount, left panel) and the cDNA yield (right panel). The Pearson correlation value is displayed above each plot. N = 5 replicates, a total of 294 cells.

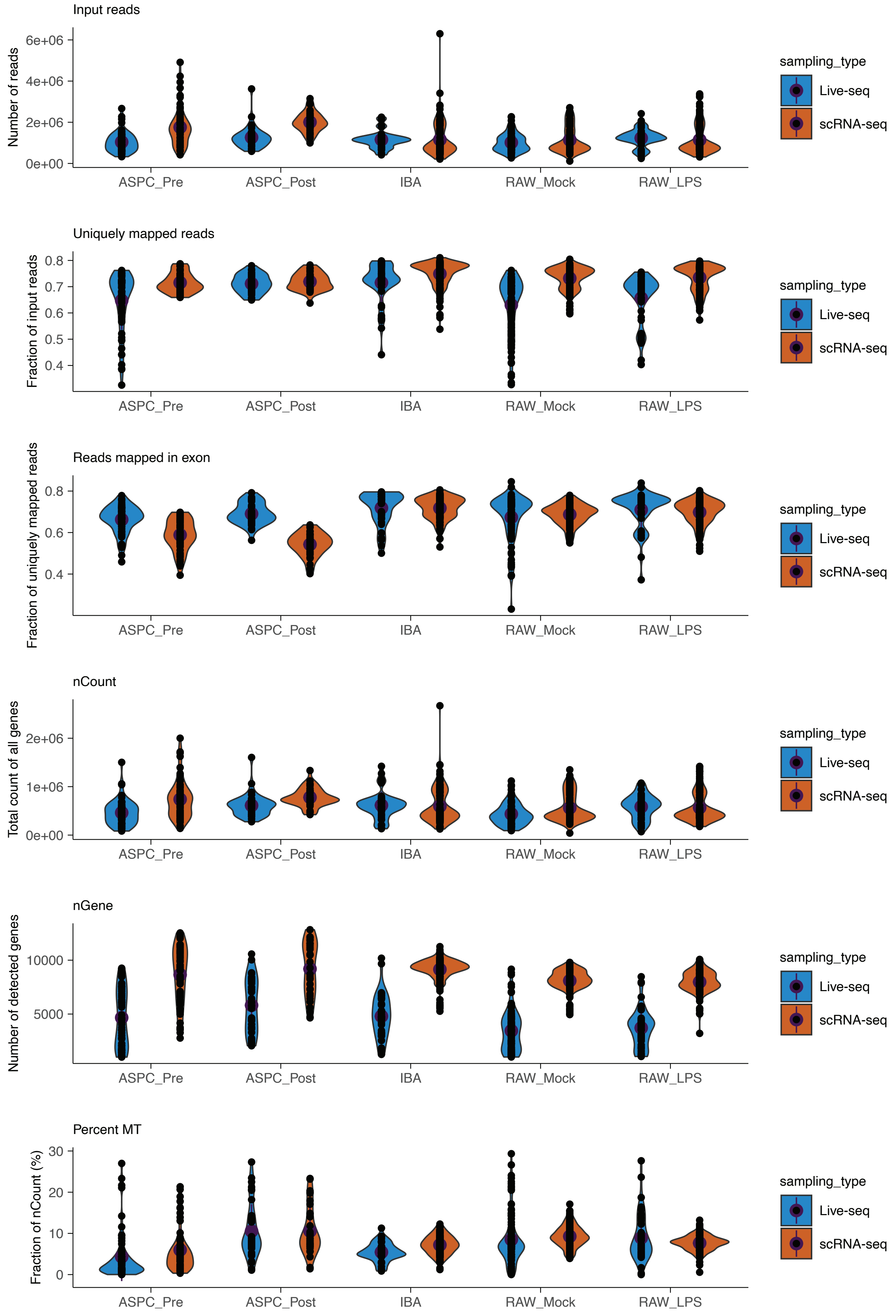

Supplementary Fig. 3. Quality control of scRNA-seq and Live-seq data plotting distinct parameters side by side as indicated above each panel. Similar to **Supplementary Fig. 2a.**

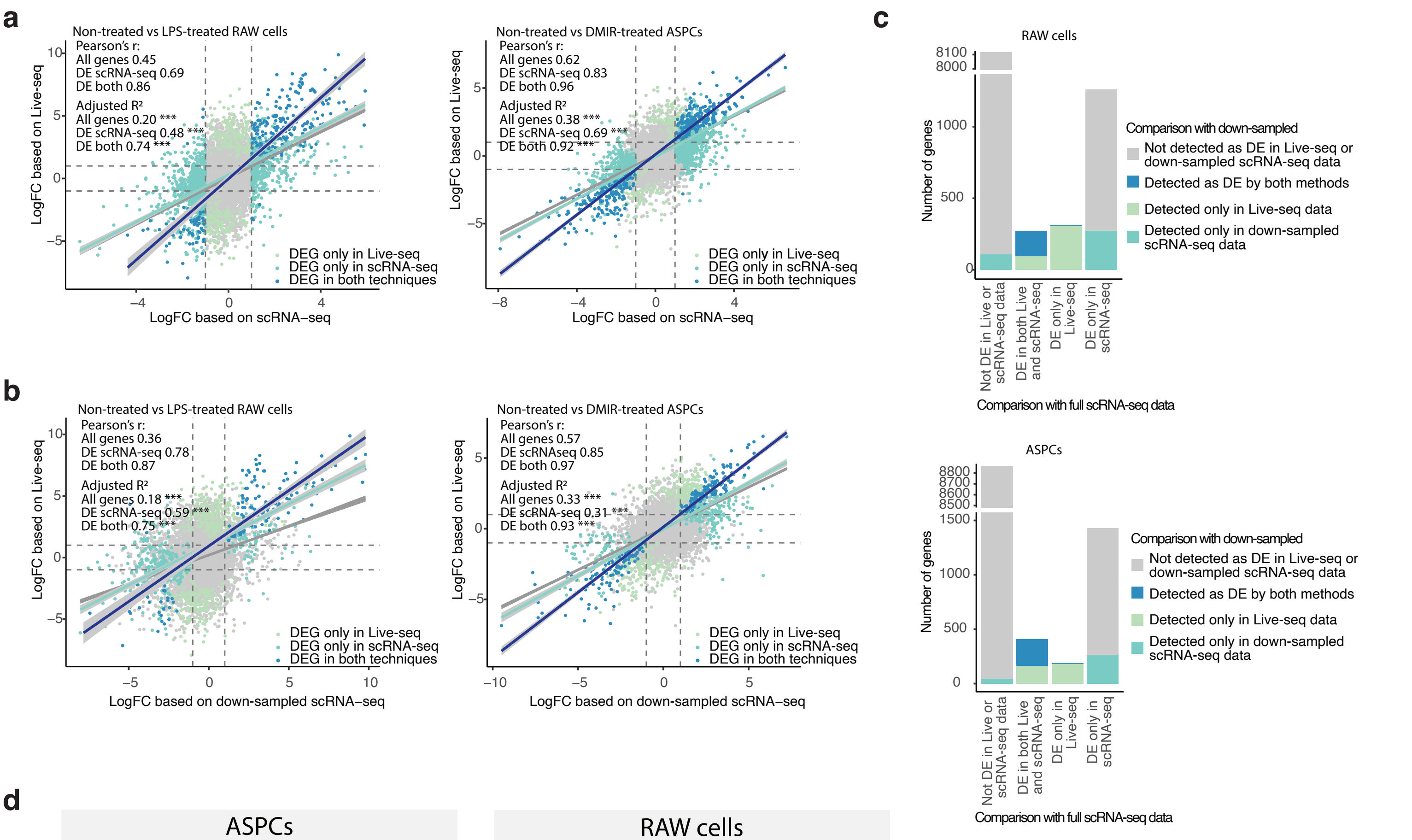

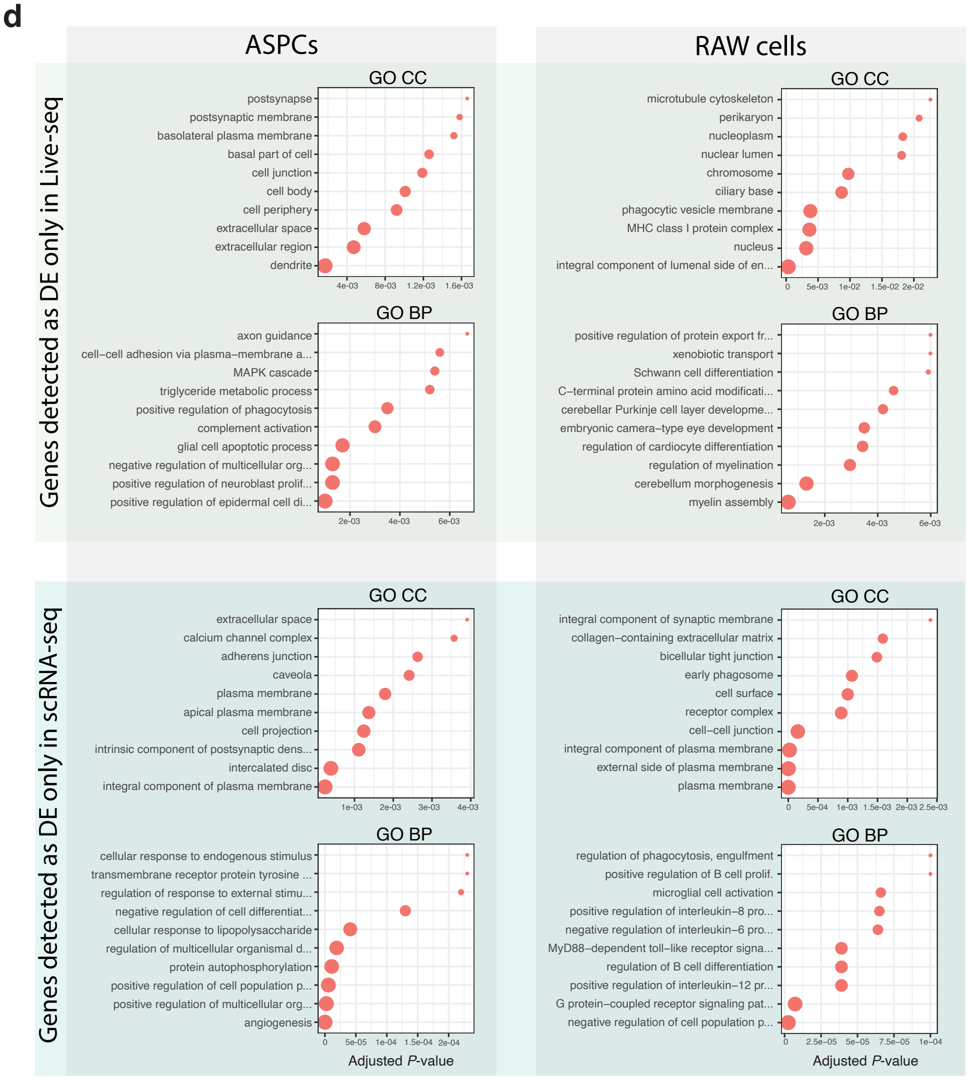

Supplementary Fig. 4. Comparison of Live-seq and scRNA-seq results per cell type, relative to Fig. 2. (**a**) Correlation of the logFC between non-treated versus treated cells of scRNA-seq vs Live-seq data (RAW cells and ASPCs). DMIR = adipogenic cocktail. (**b**) Correlation of the logFC between non-treated versus treated cells of down-sampled scRNA-seq vs Live-seq data. The scRNA-seq data were down-sampled to feature a similar library complexity (and cell number per category) as the Live-seq data. (**c**) Barplot showing the number of genes shared between the different categories: None (not identified as differentially expressed (DE) genes), Both (identified as DE both in scRNA-seq and Live-seq data), Only Live-seq (only identified as DE in Live-seq), Only scRNA-seq (only identified as DE in scRNA-seq). The x-axis shows the number of genes belonging to each category derived from the comparison between Live-seq and scRNA-seq and the colors show how these genes overlap with those derived from the comparison between Live-seq and down-sampled scRNA-seq. (**d**) Biological process (BP) and Cellular Component (CC) GO enrichment for genes detected as DE specific in either Live-seq or scRNA-seq for both ASPCs and RAW cells. The dot size corresponds to the significance of the term.

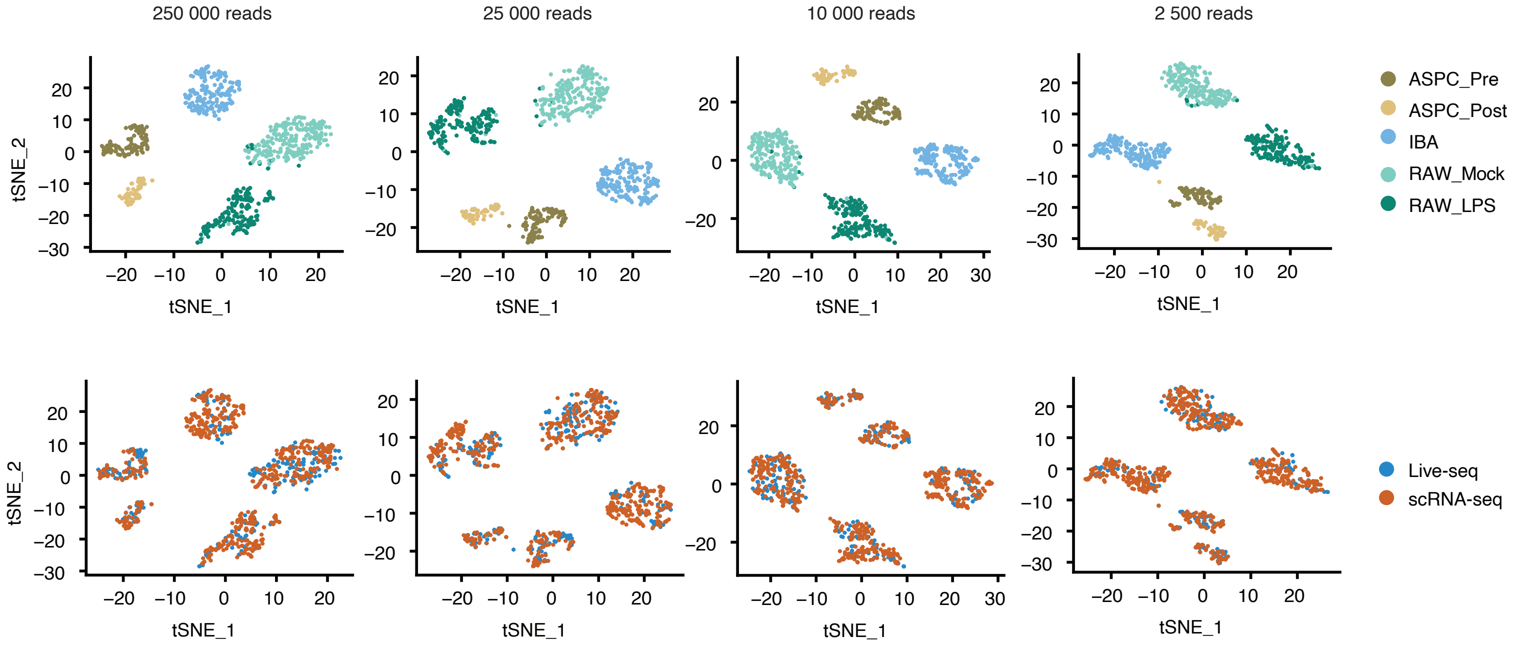

Supplementary Fig. 5. The integration of down-sampled Live-seq and scRNA-seq data, relative to Fig. 2. Evaluation of the data integration as in **Fig. 2f, g** whereby each cell was down-sampled to the indicated number of reads.

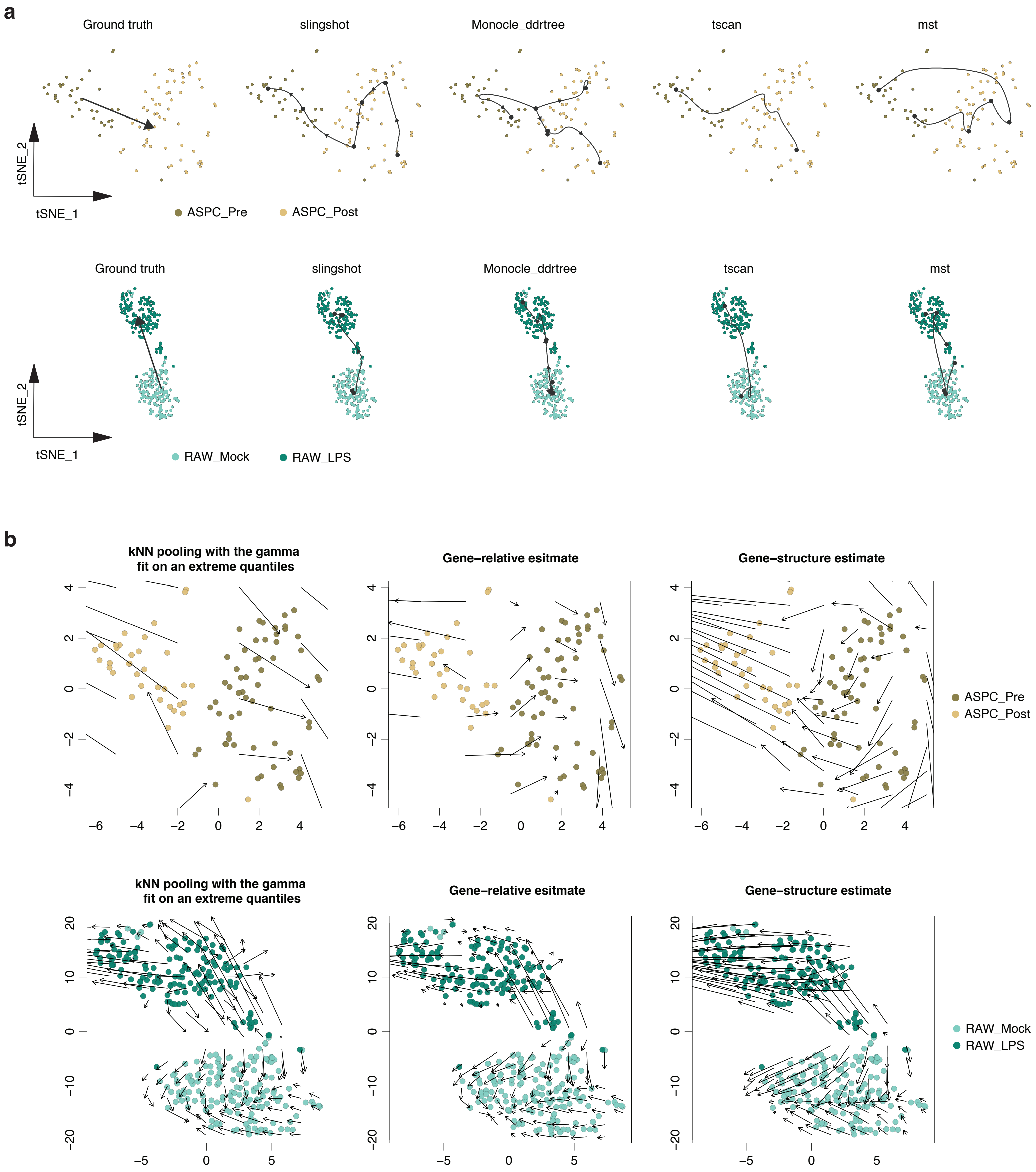

Supplementary Fig. 6. Trajectory analysis per cell type, relative to Fig. 4. (**a**) Trajectory analysis like **Extended Data Fig. 8c**, but each cell type was analyzed separately. ASPCs are shown in the upper panels, while RAW cells are shown in the lower panels. (**b**) RNA velocity analysis like **Extended Data Fig. 8d**, but each cell type was analyzed separately. ASPCs are shown in the upper panels while RAW cells in the lower panels.

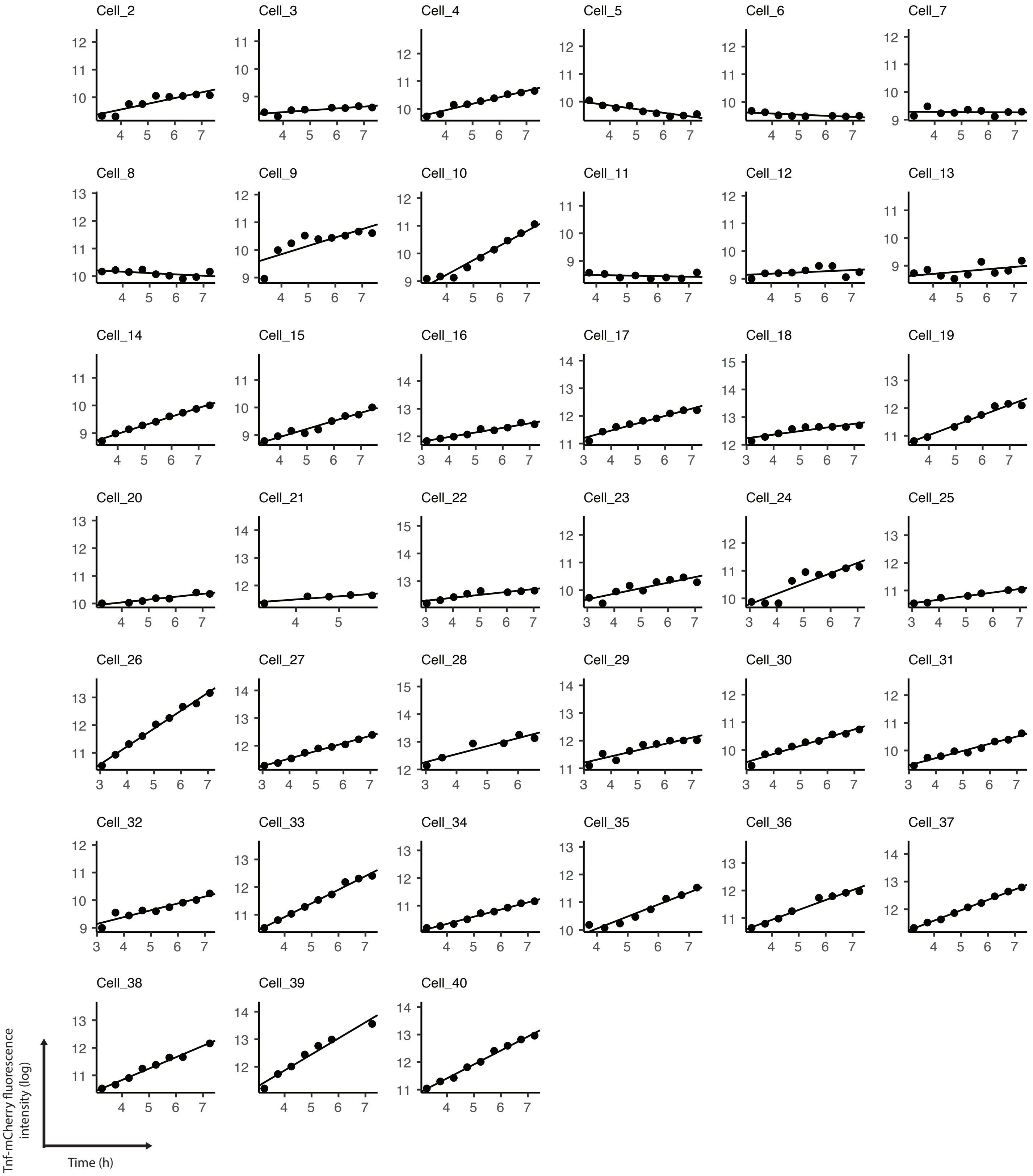

Supplementary Fig. 7. The Tnf-mCherry intensity curves of additional RAW cells shown in Extended Data Fig. 9b. The complete data showing a linear relationship between the time post-LPS treatment (within a window of 3 to 7.5 hours post-LPS treatment) and the Tnf-mCherry fluorescence intensity (log transformed) in one cell.

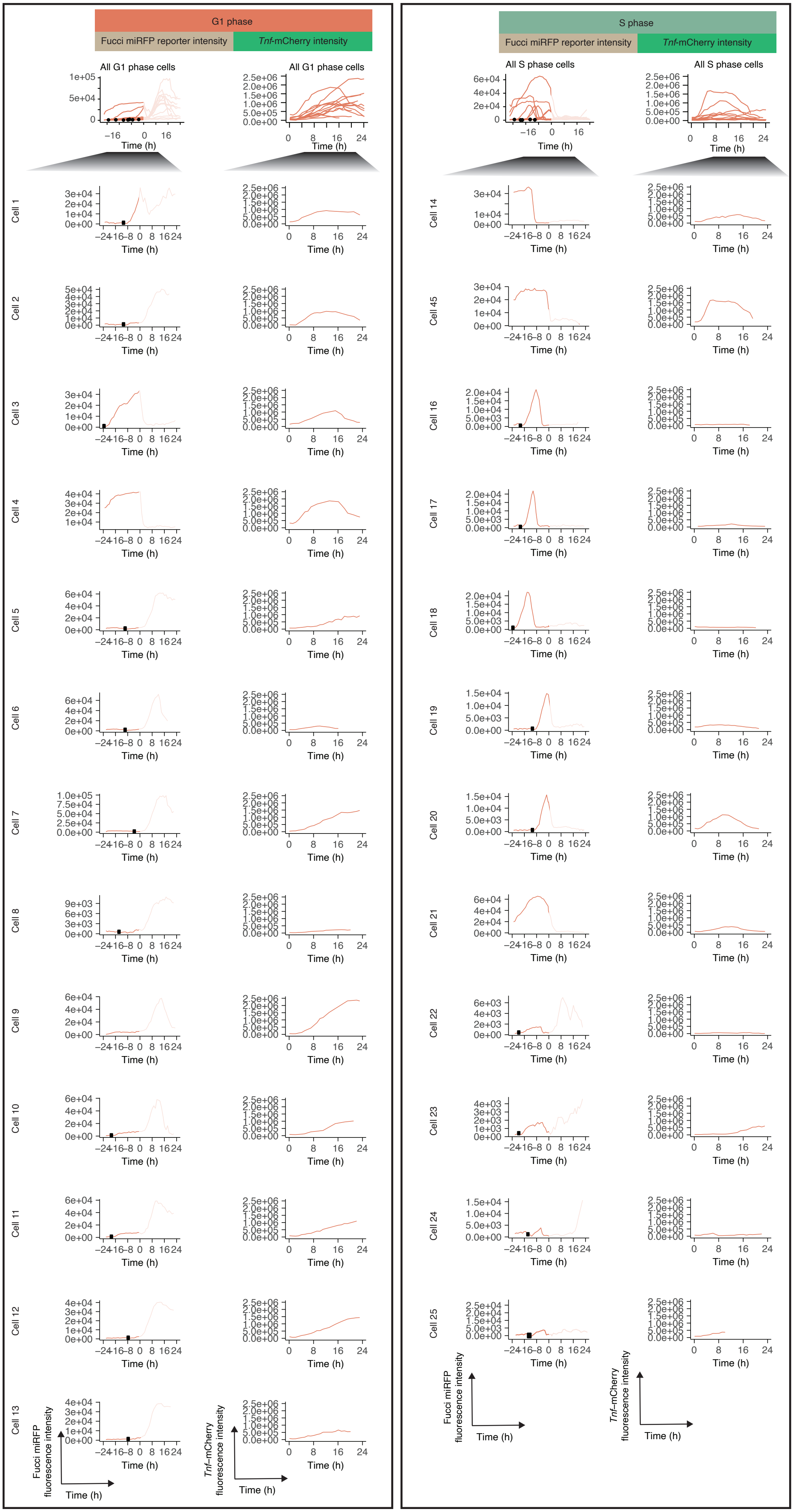

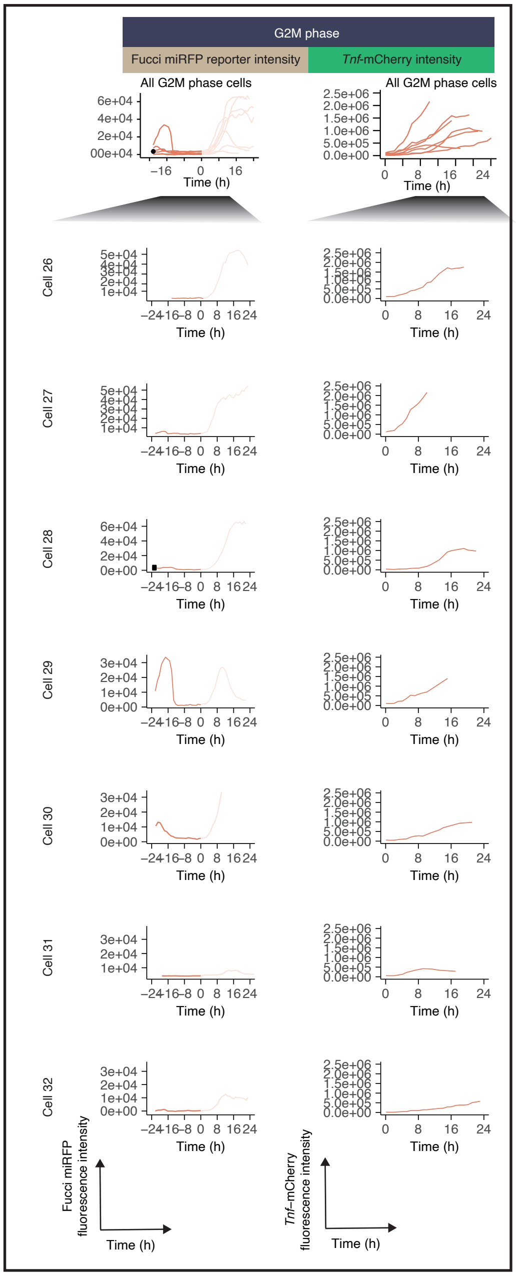

Supplementary Fig. 8. Measuring the cell cycle phase (Fucci reporter) and LPS-induced response (TNFα reporter) of RAW cells, relative to Extended Data Fig. 10b. Cell cycle phases of individual cells were determined using the Fucci reporter miRFP-hCdt1. The Fucci reporter and LPS-induced Tnf-mCherry intensities of each individual cell are shown separately, while all cells assigned to a specific cell cycle phase are merged into one meta-plot (at the top of each category) to facilitate direct comparisons. The time of LPS treatment is defined here as 0. Black dots indicate the mitosis time point. The curves of Fucci reporter after LPS treatment are not considered and are therefore shown in a lighter color. The G1/S boundary is inferred from the time point at which the Fucci reporter intensity drops. Cells that underwent mitosis but did not yet reach the G1/S boundary were annotated as G1 cells. Given the lack of a clearly discernable S/G2M boundary, cells were assigned to either the S or G2M phase based on the post G1/S boundary timing.

Supplementary Note:

Since Smart-seq2 was widely appreciated as the most RNA-seq method to detect low input of RNA at the time of method development[1], we tested whether it could amplify cDNA at the picogram scale. While successful in amplifying cDNA when the input total RNA input was above 5 pg, it failed with an RNA input of 1 pg (**Extended Data Fig. 1a**). However, 10% of the amount of cDNA reverse transcripted from 10 pg of input RNA (equivalent to 1 pg input RNA) could be amplified (**Extended Data Fig. 1a**), suggesting that the reverse transcription product from 10 pg input RNA is more than 10 times than that from 1 pg input RNA. We thus reasoned that the reverse transcription step, rather than the PCR step, is the main reason for the failure. We thus focused on the optimization of the reverse transcription process. We first compared different reverse transcriptase. Among the 5 enzymes tested, only Maxima H Minus Reverse Transcriptase produced significant output with 1 pg total RNA, but showed a similar background signal compared to the negative control (0 pg RNA) (**Supplementary Fig. 1a**). Size analysis using a fragment analyzer revealed a hedgehog-like profile of both types of cDNA, indicating an amplification of adaptor concatemers (**Supplementary Fig. 1b**). However, cDNA from 1 pg RNA showed additional peaks at around 1.1 and 1.8kb (**Supplementary Fig. 1b**). We hypothesized that 1 pg RNA could be efficiently reverse transcripted in this condition, but that this process was hindered by the large amount of adaptor concatemers. We thus tried to reduce the adaptor concatemers by modifying the template-switching oligonucleotide (TSO)[2] . Modification of the 5' end of the TSO by introducing a hairpin (hairpin-TSO) or non-natural nucleotides (iso-TSO)[2] did not generate concatemers, but did also not yield cDNA (**Supplementary Fig. 1c, Methods**), while biotin modified TSO (biotin-TSO)[3] largely reduced the concatemer background and did not compromise the cDNA yield. We then tested combinations of different amounts of the TSO, oligo-dT and the reverse transcriptase, to further reduce the background and enhance net cDNA yield (**Supplementary Fig. 1d**). The condition with 0.8 µM biotin-TSO, 1 µM oligo-dT and 0.1 µl Maxima H Minus Reverse Transcriptase (200 U/µl) outperformed (**Supplementary Fig. 1d-e**), and was thus defined as "the modified Smart-seq2 workflow" from hereon in the main manuscript. While adding UMI and barcode reduces PCR bias and enables multiplexing[4], it seems not applicable in this scenario as the cDNA yield is largely reduced (**Supplementary Fig. 1f**). We then sequenced the libraries derived from modified Smart-seq2-generated cDNA from 1 pg and 0 pg (negative control) of total RNA. For 1 pg of input RNA, the uniquely mapped rate (rate of the read mapped to the genome among total reads) and exon mapped rate (rate of reads mapped to exon among unique mapped reads) were more than 0.6, with more than 1300 genes detected, while the 0 pg RNA library showed a low uniquely mapped rate, exon mapped rate and small amount of detected genes (**Extended Data Fig. 1c**). The sequences derived from oligo-dT and TSO were overrepresented in the 0 pg RNA library (**Extended Data Fig. 1d, e**). The top 20 genes absorbed most of the mapped reads of these libraries (**Extended Data Fig. 1f**). These are likely due to sequencing errors or mis-mapping of the A/T rich region and thus were not included in downstream data analyses.

1. Ziegenhain, C. *et al.* Comparative Analysis of Single-Cell RNA Sequencing Methods. *Molecular Cell* **65**, 631-643.e4 (2017).
2. Kapteyn, J., He, R., McDowell, E. T. & Gang, D. R. Incorporation of non-natural nucleotides into template-switching oligonucleotides reduces background and improves cDNA synthesis from very small RNA samples. *BMC Genomics* **11**, 413 (2010).
3. Islam, S. *et al.* Highly multiplexed and strand-specific single-cell RNA 5' end sequencing. *Nat Protoc* **7**, 813–828 (2012).
4. Kivioja, T. *et al.* Counting absolute numbers of molecules using unique molecular identifiers. *Nat Methods* **9**, 72–74 (2012).
