# Supplementary material for: Live-seq enables temporal transcriptomic recording of single cells
Source: Nature. 2022 Aug 17;608(7924):733–40. doi: 10.1038/s41586-022-05046-9 (PMC9402441; doi:10.1038/s41586-022-05046-9)
Supplement: Supplementary file 2 — Reporting Summary. [file 41586_2022_5046_MOESM2_ESM.pdf]

nature portfolio

Corresponding author(s): Bart Deplancke

Last updated by author(s): May 7, 2022

Reporting Summary

Nature Portfolio wishes to improve the reproducibility of the work that we publish. This form provides structure for consistency and transparency in reporting. For further information on Nature Portfolio policies, see our Editorial Policies and the Editorial Policy Checklist.

Statistics

For all statistical analyses, confirm that the following items are present in the figure legend, table legend, main text, or Methods section.

n/a	Confirmed	
☐	☒	The exact sample size (*n*) for each experimental group/condition, given as a discrete number and unit of measurement
☐	☒	A statement on whether measurements were taken from distinct samples or whether the same sample was measured repeatedly
☐	☒	The statistical test(s) used AND whether they are one- or two-sided<br>*Only common tests should be described solely by name; describe more complex techniques in the Methods section.*
☒	☐	A description of all covariates tested
☐	☒	A description of any assumptions or corrections, such as tests of normality and adjustment for multiple comparisons
☐	☒	A full description of the statistical parameters including central tendency (e.g. means) or other basic estimates (e.g. regression coefficient) AND variation (e.g. standard deviation) or associated estimates of uncertainty (e.g. confidence intervals)
☐	☒	For null hypothesis testing, the test statistic (e.g. *F*, *t*, *r*) with confidence intervals, effect sizes, degrees of freedom and *P* value noted<br>*Give P values as exact values whenever suitable.*
☒	☐	For Bayesian analysis, information on the choice of priors and Markov chain Monte Carlo settings
☒	☐	For hierarchical and complex designs, identification of the appropriate level for tests and full reporting of outcomes
☐	☒	Estimates of effect sizes (e.g. Cohen's *d*, Pearson's *r*), indicating how they were calculated

Our web collection on statistics for biologists contains articles on many of the points above.

Software and code

Policy information about availability of computer code

Data collection	Basecalls performed on NextSeq 500/HiSeq 4000 result using bcl2fastq v2.19.1
Data analysis	STAR (2.6.1c); HTseq (0.10.0); R (version 3.5.0); ggplot2 (version 3.2.1); Seurat R package (v3.0); EnrichR(3.0); ClusterProfiler(3.14.3); msigdbr (7.1.1); org.Mm.eg.db (version 3.10.0); dynverse (0.1.2); velocyte(0.6);

For manuscripts utilizing custom algorithms or software that are central to the research but not yet described in published literature, software must be made available to editors and reviewers. We strongly encourage code deposition in a community repository (e.g. GitHub). See the Nature Portfolio guidelines for submitting code & software for further information.

Data

Policy information about availability of data

All manuscripts must include a data availability statement. This statement should provide the following information, where applicable:

- Accession codes, unique identifiers, or web links for publicly available datasets
- A description of any restrictions on data availability
- For clinical datasets or third party data, please ensure that the statement adheres to our policy

All Live-seq and scRNA-seq data are available in the Gene Expression Omnibus (GEO) with accession number GSE141064.

Field-specific reporting

Please select the one below that is the best fit for your research. If you are not sure, read the appropriate sections before making your selection.

☒ Life sciences ☐ Behavioural & social sciences ☐ Ecological, evolutionary & environmental sciences

For a reference copy of the document with all sections, see nature.com/documents/nr-reporting-summary-flat.pdf

Life sciences study design

All studies must disclose on these points even when the disclosure is negative.

Sample size	There are 10 IBA cells and Hela cells processed through Live-seq sampling in alternative fashion. In addition, 588 Live-seq samples were prepared across five experimental replicates, with each of them containing both single and sequential sampling events. All the data from these 588 cells were used for the analyses presented in Figures 2-5. 554 cells were processed using conventional scRNA-seq as part of three experimental replicates and the data linked to these cells are presented in Figures 2-5. To evaluate the potential molecular perturbation of cytoplasmic sampling, scRNA-seq data of IBA cells 1 hour (49 cells) and 4 hours (43 cells) post extraction were generated, as well as of control cells that were not subjected to such sampling (70 cells) . To evaluate the post-extraction viability of ASPC (N=33) and IBA (N=37) cells, the cells were stained between 2 and 4 hours after extraction using a LIVE/DEAD® Cell Imaging Kit 488/570 (Invitrogen), and following the manufacturer's protocol. To evaluate the post-extraction viability of RAW264.7 cells, the extracted cells (N= 72) were monitored by time-lapse microscopy. We analysed 77 extracted cells, 122 LPS-stimulated cells that were not extracted, and 23 cells that were not extracted and not stimulated with LPS. Statistic tests were performed for all the analyses as indicated in the Figure legends and methods accordingly. While the sample size for Live-seq to couple initial transcriptome with subsequent LPS response is insufficient to explain the full variant at each single gene level, we have discussed the limitation of Live-seq at the current stage in the result and discussion section of the main text.
Data exclusions	No data is excluded, except for RNA-seq samples showing low quality were filtered out, with quality cutoffs being: i) the number of genes <1000, ii) the mitochondrial read ratio >30%, or iii) the uniquely mapped rate <30%. This is described in the methods.
Replication	Live-seq samples and conventional scRNA-seq were prepared across five and three replicates, respectively. Enhanced Smart-seq2 optimization was performed with three replicates. The calculation of cell volume is performed per cell base (N=277 for ASPC and N=500 for IBA and RAW). Time-lapse monitoring of mCherry expression was performed with three replicates. Cell viability after extraction was checked with more than 3 replicates. These replicates successfully show consistent results.
Randomization	No randomization was applied as only cell lines and no human or animal subjects were used.
Blinding	The imaging of lipid accumulation in ASPC was performed blindingly. Blinding is not necessary for other analysis as they are quantitative and no subjective interpretation is required.

Reporting for specific materials, systems and methods

We require information from authors about some types of materials, experimental systems and methods used in many studies. Here, indicate whether each material, system or method listed is relevant to your study. If you are not sure if a list item applies to your research, read the appropriate section before selecting a response.

Materials & experimental systems

n/a	Involved in the study	
☒	☐	Antibodies
☐	☒	Eukaryotic cell lines
☒	☐	Palaeontology and archaeology
☐	☒	Animals and other organisms
☒	☐	Human research participants
☒	☐	Clinical data
☒	☐	Dual use research of concern

Methods

n/a	Involved in the study	
☒	☐	ChIP-seq
☒	☐	Flow cytometry
☒	☐	MRI-based neuroimaging

Eukaryotic cell lines

Policy information about cell lines

Cell line source(s)	RAW264.7, 293T and Hela cells were obtained from ATCC. RAW264.7 cells with Tnf-mCherry reporter and relA-GFP fusion protein (RAW-G9 clone) were generated and kindly provided by Dr. Iain D.C. Fraser (NIH). The IBA cell line derived from the stromal vascular fraction (SVF) of interscapular brown adipose tissue of young male mice (C57BL6/J) was generated and kindly provided by Prof. Christian Wolfrum's laboratory (ETHZ). Primary ASPCs were isolated from subcutaneous fat tissue of C57BL/6J mice.
Authentication	None of the cell lines used were authenticated.

Mycoplasma contamination	The cell lines were not tested for mycoplasma contamination.
Commonly misidentified lines (See ICLAC register)	No cell line is misidentified to our knowledge.

Animals and other organisms

Policy information about studies involving animals; ARRIVE guidelines recommended for reporting animal research

Laboratory animals	All mice used in the remaining experiments were wild-type C57BL/6J males and females (median of age: 8.7 weeks ranging 7-11 weeks, median of weight: 22.5 g ranging 20.4-23.4 g). Mice were housed in SFP condition at 22 °C with 40%-60% humidity and a 12 light/12 dark cycle.
Wild animals	No wild animals were used in the study.
Field-collected samples	The study did not involve field-collected samples.
Ethics oversight	All mouse experiments were conducted in strict accordance with the Swiss law, and all experiments were approved by the ethics commission of the state veterinary office (license number VD 3406, valid from 14/10/2018 to 14/01/2022).

Note that full information on the approval of the study protocol must also be provided in the manuscript.
